# The Possible Positive Mechanisms of Pirenoxine in Cataract Formation

**DOI:** 10.3390/ijms23169431

**Published:** 2022-08-21

**Authors:** Phit Upaphong, Chanisa Thonusin, Janejit Choovuthayakorn, Nipon Chattipakorn, Siriporn C. Chattipakorn

**Affiliations:** 1 Department of Ophthalmology, Faculty of Medicine, Chiang Mai University, Chiang Mai 50200, Thailand; 2 Neurophysiology Unit, Cardiac Electrophysiology Research and Training Center, Faculty of Medicine, Chiang Mai University, Chiang Mai 50200, Thailand; 3 Cardiac Electrophysiology Unit, Department of Physiology, Faculty of Medicine, Chiang Mai University, Chiang Mai 50200, Thailand; 4 Center of Excellence in Cardiac Electrophysiology Research, Chiang Mai University, Chiang Mai 50200, Thailand; 5 Department of Oral Biology and Diagnostic Sciences, Faculty of Dentistry, Chiang Mai University, Chiang Mai 50200, Thailand

**Keywords:** anticataract, antioxidant, Catalin, eye, Kary uni, PRX

## Abstract

Cataract is the leading cause of blindness worldwide. A diverse range of medication has been invented to prevent or treat cataract. Pirenoxine (PRX), a drug with strong antioxidant properties, has been used topically to treat cataract, and there is much evidence to demonstrate the beneficial effects of PRX on lens opacity from in vitro and in vivo models. In clinical use, PRX has been prescribed worldwide by ophthalmologists for over six decades; however, there is still controversy with regard to its efficacy, and thus PRX remains an off-label use for cataract treatment. This comprehensive review summarizes and discusses evidence pertinent to the mechanisms of PRX and its efficacy mainly on cataract models. The issues that have been deemed uncertain over the six-decade use of PRX are examined. The information summarized in this review should provide insights into contriving novel approaches for the treatment of cataract.

## 1. Introduction

Cataract is the major cause of global blindness in people aged 50 and older. The number of the world population with blinding cataract in 2020 was estimated to be about 15.2 million cases [1]. Interestingly, the projected number of cases with blinding cataract has been investigated only in the Chinese population [2]. That study reported that cataract blindness will be increased from 13.3 million cases in 2020 to 16.6 million cases in 2050. Moreover, the current global median cataract surgical rate (CSR) is only 1700 operations per million cases per year [2]. Age-related cataract is one type of cataract that can be defined as a lens opacity in people aged >50 years old without direct mechanical, chemical, or radiation insults [3]. Ageing-induced alterations of the lens can be a result of both enzymatic and non-enzymatic changes [3,4,5,6,7,8]. The latter changes, including conformational changes, loss of solubility and aggregation of protein, oxidative damage to various substances, increased Ca^2+^ level, and electrolyte/osmotic dysregulation, are the most common alterations observed in age-related cataract [3,5,7,8]. Those alterations in the lens increase light scattering, leading to lens opacity and the reduction of visual acuity (VA) [7]. Crystallins, consisting of α, β, and γ subtypes, are soluble proteins and the major components of the lens [7,8]. These proteins can remodel during a lifetime in order to repair the lens; however, the repairability of the lens significantly decreases with ageing [8]. Several factors lead to conformational alterations of the lens proteins, two of which are: (1) increased oxidation of cysteine, which is composed of two S-containing amino acids bound with a disulfide bridge, and (2) increased nonspecific hydrophobic interactions [9].

Although surgery is the definitive therapy to treat cataract and restore vision, there is a limited number of ophthalmologists worldwide, and this is one of the major barriers to treatment [10]. As a result, the CSR in developed countries is over 10,000 operations per million cases per year, while it is less than 500 operations per million cases per year in some low-income countries [2]. Pharmacological treatment is more accessible and safer for patients with cataract than the surgical approach [10,11,12]. Additional medications, such as taurine and lanosterol, have been invented to either prevent or treat lens opacification or cataract, similarly to pirenoxine. However, both medications have different mechanisms to treat opacification of the human lens. Taurine is reported to have an antioxidative effect for reducing lens opacification [13], but lanosterol attenuated lens opacification via disaggregating protein in cataractous lenses [14]. However, there are still no standard drugs for cataract therapy because of the controversial findings in their efficacy from clinical trials.

Pirenoxine (PRX), also called pirfenossone and pyrphenoxone, is a xanthomatin, a visual pigment found in the eye of several insects with a chemical composition of 1-hydroxy-5-oxo-5H-pyrido-[3,2-a]-phenoxazine-3-carboxylic acid. Several previous studies reported that PRX is a drug exerting a strong antioxidant effect with the capacity to ameliorate lens opacity [12,15,16,17,18,19]. PRX was firstly introduced in 1958 to prevent cataract [20,21,22,23]. Its efficacy in the treatment of age-related cataract was approved based upon the evidence from three studies [20,21,22], following which it has been widely used for cataract treatment in Japan [24]. PRX appeared on the market under the tradename of Catalin^©^ and Kary Uni^©^, both in the forms of eyedrops containing 0.005% of PRX. Catalin is formulated in tablets and needs to be dissolved in solvent before being used. In contrast, Kary Uni can be instantly applied. Despite being available worldwide for over six decades, its efficacy is still controversial [12,15,16,17,18,24,25,26,27,28,29], and PRX has been mainly an off-label use for age-related cataract, its pathophysiology mainly being a result of increased oxidative stress in the lens [17,30]. Even though PRX is widely prescribed for age-related cataract, several researchers have shifted their focus to the benefits of PRX on diabetic cataract prevention and treatment [18,31,32,33,34,35,36].

This comprehensive review aims to summarize and discuss previous evidence regarding the mechanisms of PRX and its efficacy on cataract models. The issues that have been deemed uncertain over the six-decade use of PRX are examined. This review article will describe the proposed mechanisms, clinical applications, and the future perspective of PRX usage.

Previous original articles in English were searched via PubMed using the following keywords: (“phenoxazin*” or “pirenoxine” or “pyrphenoxone” or “Kary uni” or “Catalin”) and “eye”. All relevant articles from in vitro to clinical studies from 1955 to March 2022 were retrieved. The references included in each relevant article were thoroughly screened in a further manual search. 

## 2. Effects of Pirenoxine on Age-Related Cataract: Evidence from In Vitro, Ex Vivo, In Vivo, and Clinical Studies

Cataract is a multifactorial disease involving genetics, ageing, oxidative stress, radiation, nutritional deficiency, metabolic disorders, diabetes, trauma, and specific chemical substances [3,5]. During the human lifespan, the lens is exposed to oxidative stress via both endogenous routes, including mitochondrial respiration and oxidative burst from macrophages, and exogenous routes including ultraviolet light (UV), tobacco smoke, metals, and drugs [3,5,6]. The notorious effects of UV radiation to the eye are widely known; however, the 193 nm argon–fluoride excimer laser (the UVC range), which also causes oxidative stress to the cornea, is now used in laser refractive surgeries [37]. An in vivo study demonstrated that the excimer laser was a possible risk of cataract, as indicated by the alterations of refractive index and molecular weight of lens proteins [38]. The association between laser refractive surgeries and early cataract has been shown in clinical studies [39,40].

Although several causes of age-related cataract cannot be controlled, previous studies demonstrated the protective effects of PRX against cataract from various factors, including selenite, calcium, UVC, ferric (Fe^3+^), hemoglobin (Hb), and stimulated macrophages [15,16,17,19,25]. The effects of PRX on age-related cataract from in vitro studies are summarized in Table 1, and those from ex vivo, in vivo, and clinical studies are summarized in Table 2. The following information summarizes the effects of PRX on different models of age-related cataract, including calcium dysregulation, oxidative stress, selenium, ultraviolet radiation, and quinone.

### 2.1. Effects of Pirenoxine on Calcium Dysregulation-Induced Age-Related Cataract

With ageing, Ca^2+^-ATPase activity that plays an important role in lens’ calcium regulation decreases, resulting in an increased influx of Ca^2+^ [4]. Moreover, a decreased calcium-binding capacity of lens lipids leads to an elevation of intracellular Ca^2+^ [4]. Calcium can induce cataract formation by: (1) promoting α-crystallin aggregation, (2) stimulating protease enzymes in the lens, and (3) reducing the chaperone activity involved in protein folding. All of these mechanisms lead to proteolysis, light scattering, and opacity of the lens [4,41].

An ab initio study theoretically proposed the possibility of PRX as a Ca^2+^-chelator indicated by the observation of a binding site of PRX to Ca^2+^ [15]. In vitro studies revealed that PRX decelerated Ca^2+^-induced lens opacification, as indicated by a deceleration in protein particle turbidity measured by spectroscopy [15,16]. On the other hand, an in vivo study reported a neutral effect of PRX on Ca^2+^ level [25]. This neutral finding could potentially be due to the low dose and short duration of PRX used in that study.

Calpain is one of the calcium-dependent cysteine proteases that is involved in cataract formation, especially the cortical type [4]. Under Ca^2+^ overload conditions, the activity of calpain was found to increase, leading to α-and β-crystallin proteolysis in the lens, and eventually lens opacification [4]. Despite the Ca^2+^-chelating effect [15,16] of PRX, PRX itself cannot compete with Ca^2+^ to bind to calpain; therefore, it could not prevent m-calpain (calpain II)-induced degradation of lens protein, while ethylenediamine tetraacetic acid (EDTA) and calpain inhibitor E64 did [15]. These findings suggested that PRX only plays a role in the non-enzymatic Ca^2+^-induced cataract. Nevertheless, no in vivo nor clinical studies have affirmed these findings.

### 2.2. Effects of Pirenoxine on Oxidative Stress-Induced Age-Related Cataract

Oxidative stress is a major factor of cataractogenesis [3,6]. Production of reactive oxygen species (ROS) initiates all types of cataract: cortical, nuclear, and posterior subcapsular [30]. Glutathione (GSH) and ascorbate are the main oxidant scavengers of the lens [42]. GSH maintains the lens transparency as a result of several mechanisms including: (1) protection of the thiol groups of crystallins in the reduced form, therefore preventing disulfide cross-link formation, (2) regulation of electrolytes by preservation of the thiol groups in the lens membrane, and (3) counteraction of hydrogen peroxide (H_2_O_2_)-induced oxidative damage [13]. With ageing, the amount and activity of lenticular antioxidants, particularly in the nucleus, decline [8]. GSH levels and GSH transport to the lens core, and superoxide dismutase (SOD) and catalase (CAT) enzyme activity in the lens significantly decrease [8].

Although ascorbate is considered to be an antioxidant, it can be a prooxidant when free iron is present and GSH is absent [6]. Iron overload has been reported to be a cause of cataract [43]. In the physiological condition, Fe^2+^ can be oxidized by H_2_O_2_ to become Fe^3+^, and then formulate a hydroxide ion (OH^−^) and a hydroxyl radical (OH^•^). Ascorbate changes Fe^3+^ to Fe^2+^, and Fe^2+^ expedites the production of ROS, resulting in crosslinked peptide formation [6]. Then, the induction of iron/ascorbate simulates the conditions of oxidative stress load [6,17]. This induction acts as the physiological change during ageing, as indicated by an increase in iron level in the cataractous lens of aging people [44]. According to in vitro and in vivo studies [17], PRX prevented oxidative damage of the lens after it had been induced with either Fe^3+^ or hemoglobin (Hb), as shown by a reduction in the lipid peroxidation byproducts similar to that of the baseline level. The mentioned lipid peroxidation byproducts are lipid hydroperoxide and malondialdehyde (MDA), which is measured by the thiobarbituric acid (TBA) test. Likewise, another in vitro study revealed that PRX decelerated MDA in serum after induction with Fe^2+^ [45].

Diquat is a herbicide involving cyclic reduction–oxidation reactions [46]. With the potential to produce superoxide radicals and deplete nicotinamide adenine dinucleotide phosphate (NADPH), diquat also causes an increase in oxidative stress [46]. An in vitro study showed that PRX decreased lipid peroxidation following the intravenous (IVT) injection of diquat [17]. PRX also counteracted the oxidative burst induced by n-formyl methionyl-leucylphenylalanine (fMLP)-stimulated macrophages [17].

Xanthine oxidase (XO) is an enzyme converting xanthine (X) to uric acid. This process also reduces O_2_ and generates ROS production. An in vitro experiment generating ROS production from the X/XO system demonstrated that PRX prevented lipid peroxidation [17]. However, this effect of PRX was independent of the inhibition of the X/XO system since the level of superoxide and urate were unchanged [17].

Most of the findings from in vitro and in vivo studies discovered the positive impact of PRX on increased GSH levels in the lens [18,19,47,48], and the maintenance of Na^+^/K^+^ channels via an oxidative protective mechanism of membrane cationic pumps [34,49]. However, these positive effects were not observed in one in vivo study [25], a controversial finding that could be explained by the lower dose and shorter duration of PRX used in that study. In addition to GSH in the lens, an in vivo study showed that not only SOD and CAT activity in the lens, but also serum GSH, SOD, and CAT levels were increased after the administration of topical PRX [19]. Furthermore, the level of MDA was found to have declined in the serum [19]. All of these findings indicated that a significant amount of topical PRX application could be absorbed through the systemic circulation. However, the information regarding the systemic effects of PRX remains limited.

### 2.3. Effects of Pirenoxine on Selenium-Induced Age-Related Cataract

Selenium-induced cataract in animal models causes an alteration in the lens protein profile that is similar to ageing-induced cataract; thus, selenite cataract is a good representative model of human age-related cataract [50]. The mechanism of selenium-induced cataratogenesis is attributed to: (1) decreased calcium-ATPase activity and increased calcium-induced proteolysis [15,51], and (2) stimulated ROS production and decreased GSH levels [51,52]. In vitro studies revealed that PRX attenuated selenite cataract via chelating Se ions and subsequently by decreasing the degradation of crystallin proteins [15,16].

An ab initio study theoretically confirmed the possibility of Se chelation by PRX [15]. That study demonstrated that six Se ions could be bound to a molecule of PRX in a concentration-dependent fashion [15]. Se ions were more likely to be attracted to PRX rather than to the thiol groups of lens protein, and therefore PRX prevented further changes in the lens protein [15]. Conversely, in vivo experiments showed that pre-treatment with PRX in topical, IVT, and subcutaneous (SC) forms failed to decelerate selenite-induced lens opacity [15,25]. Although absorption of lipophilic drugs through the cornea is better than that of hydrophilic drugs [53], a prior study reported that PRX in both solution and liposomal form could not decelerate selenite cataract in rats [25]. The inconsistent results between in vitro and in vivo studies could be due to the inadequate dosage of PRX used in the in vivo models [15]. In support of this possibility, a previous study proposed that increasing PRX dosage might provide a positive effect, since decreased lens opacity remained present in the first three days after selenite injection [15].

### 2.4. Effects of Pirenoxine on Ultraviolet (UV) Radiation-Induced Age-Related Cataract

UVA and a small portion of UVB that passes through the cornea are then absorbed by the lens [8,54]. For this reason, exposure to UV light causes cataract via photo-damaging effects, as well as inducing cross-linking, oligomerization, and proteolysis of crystallins [42]. An optimal dose of PRX could protect the lens against UVC by decelerating crystallin protein degradation, resulting in a decrease in the lens opacity [15]. 

The solvents of Catalin, called ‘cataV’—inactive ingredients of Catalin—have been reported to exert a lens-protective effect against UVC, as indicated by a deceleration in the degradation of crystallins and lens opacity when ‘cataV’ was used separately [15]. The aqueous dissolution of Catalin consists of polyvinyl alcohol, succinic acid, sodium succinate, sodium chloride, sodium edetate, and benzakonium chloride [55,56]. In contrast, an in vitro study showed that cataV has no effect on the serum lipid peroxidation induced by Fe^2+^ [45], suggesting that cataV has no antioxidant properties. The positive effect of cataV on lens transparency may be due to the Ca^2+^-chelation effect of sodium edetate [57]. Interestingly, taurine that is added in the dry power of Catalin from some manufacturers [55] also possesses antioxidant properties and possibly decelerates cataract formation [58]. However, the protective effect of both PRX and cataV were not detected after UVB irradiation [15]. The possible explanations of the null effect of PRX in the condition of UVB irradiation might be: (1) the insufficient dosage of PRX, and (2) the action of 3-hydroxykynurenin, which occurs after PRX reacts with proteins after UVB exposure [15]. The substance 3-hydroxykynurenin can escalate protein aggregation, resulting in lens opacity [15].

### 2.5. Effects of Pirenoxine on Quinone-Induced Age-Related Cataracts

Interestingly, PRX was firstly invented based on quinonic theory, in which it was proposed that endogenous quinone could contribute to cataractogenesis [20,21,22]. Although this theory is not now accepted [59], exogenous quinones such as naphthalene have been used in a simulation of age-related cataract [60]. Exogenous substances exert a cataractogenic effect via two mechanisms: (1) interaction with thiol groups of β- and γ-crystallins, leading to formation of insoluble colored proteins as observed in aged lens [51], and (2) ROS generation, leading to a decrease in GSH level. An in vitro study revealed that PRX competed with quinonic substances, in which PRX could bind to the thiol groups of the lens proteins, preventing further oxidation [22]. In addition, PRX decelerated cataract formation induced by intraperitoneal (IP) injection of benzoquinone acetic acid [22].

### 2.6. Effects of Pirenoxine on the Natural Progression of Cataract

In vivo studies revealed that PRX decelerated lens opacity and slowed the progression of age-related cataract [27,28]. Of these findings, one study showed that the effects of PRX were noticeable after 81.6 days of treatment [27]. Interestingly, these beneficial effects were more prominent in the cortical region of the lens, especially in younger models [27]. However, results from clinical studies are controversial. 

Three previous clinical trials discovered the positive effect of PRX on the deceleration of lens opacity and cataract progression [12,21,61]. Microbioscopic lens images from a Scheimpflug camera confirmed a reverse in lens opacity after only one month of PRX treatment, especially in the cortical and posterior subcapsular layers [61]. The peak effect of PRX was observed after 18 months of continuous treatment [12]. Interestingly, PRX was efficient in both presenile (cataract before the age of 50 years) and age-related cataract, but the change was more prominent in those younger than 59 years old [12]. Nonetheless, the methodology of this study is questionable [12]. Not only did it decelerate structural changes in the lens, but PRX also impeded VA loss from age-related cataract for 8 to 24 months of observation [21]. In contrast, a few clinical studies revealed that PRX was ineffective for cataract prevention [26,29]. One study showed that PRX seemed to be significantly inferior to benzyl alcohol in terms of improved lens opacity, increased VA, and a reduction in the need for cataract surgery [26]. Interestingly, a large clinical trial reported that PRX had no effect on the delayed progression of cataract and the improvement of VA [29]. In that trial [29], the contralateral eye of the same individual was used as a control, which was unlike other studies. The use of a contralateral eye control could minimize the impact of environmental cataractogenic factors among the participants.

All of those previous results suggested that the positive effect of PRX was evident in a study that included only the cortical opacity [12]. In contrast, the neutral effect of PRX was observed in a study that included unlimited patterns of lens opacity [26,29]. Hence, PRX may only have positive effects on the cortical type of cataract. Further clinical studies are warranted.

## 3. Effects of Pirenoxine on Diabetic Cataract

Diabetes mellitus is positively associated with overall incidence of cataract and is a cause of pre-senile cataract [62]. Lens opacity in diabetic cataract is attributed to sorbitol accumulation mediated by aldose reductase (AR), ROS generation, and dysfunction of the Na^+^/K^+^ pump and calcium-ATPase, leading to increased intracellular Na^+^ and Ca^2+^ levels and increased osmotic stress [41,62,63]. Several in vitro and in vivo studies demonstrated that PRX decelerated or reversed the lens opacity of hyperglycemic models, conditions that were induced by either hyperglycemic solutions or alloxan—an agent that selectively damages the beta-cells of the pancreas [18,31,32,33,34,35,36]. Interestingly, only 0.001% of PRX, which is five times lower than the prescribed concentration, seemed to be effective in reversing lens opacity in the diabetic model [32].

The effects of PRX on diabetic cataract are summarized in Table 3. The proposed mechanisms of PRX in both treatment and prevention of diabetic cataract include: (1) the interference with lens glucose metabolism mediated by AR in the polyol pathway via NADPH oxidation, resulting in inhibition of sorbitol synthesis and a reduction of further osmotic damage [33,47,64], (2) regulation of Na^+^ and K^+^ levels in the lens via a normalization of the cationic pump in the lens capsule [34,49], (3) protection of the lens protein by binding to the sulfhydryl group [18], and (4) antioxidative effects via an increase in GSH level [18]. With the similar mechanisms between the conversion of glucose to sorbitol and galactose to galactitol, it is highly suggestive that PRX also interferes with galactose metabolism. For this reason, whether induction was by an excessive glucose or galactose diet, the studies into the impact of PRX on diabetic cataract showed comparable results [32].

Hypoglycemic effects of PRX were confirmed from in vivo studies [35,65]. The administration of PRX via subcutaneous (SC), intravenous (IV), or IP routes in animal models demonstrated hypoglycemic effects in a dose-dependent manner [35,65]. These findings suggested that the hypoglycemic effects of PRX were similar to those of biguanides, but a stimulation of beta-cells for further insulin release was less likely to be due to the effect of PRX [35]. The hypoglycemic effect lasted one and four hours after the introduction of PRX by the IV and SC route, respectively [65]. Concerning the drug concentration in those previous studies, the effect of prescribed PRX (only 0.005% of concentration) on lens transparency maintenance could not be mediated by PRX-induced improved hyperglycemia. That possibility was supported by a clinical study in which the blood sugar level of the patients with congenital cataract was not altered after treatment with PRX [29].

The effect of PRX on causing the reverse of lens opacity in diabetic cataract was consistent among in vitro and in vivo studies [18,31,32,33,34,36,65]. This finding was dissimilar to those with the non-diabetic cataract, which increased the controversy as to whether PRX had the potential to reverse lens opacity or not [15,16,18,25,26,27,28,29]. Interestingly, the effect of PRX on the reverse of diabetic cataract could be explained by the temporary change of lens opacity in diabetic cataract, since a clinical study showed that good glycemic control itself could also ameliorate early-stages of diabetic cataract [62]. 

## 4. Effects of Pirenoxine on Congenital Cataract

Tryptophan (Trp) is an essential aromatic amino acid. Deprivation of Trp results in the decline of: (1) β-crystallin synthesis, (2) kynurenine, one of the physical UVA filters in the lens nucleus, and (3) the activity of indoleamine-2,3-dioxygenase, one of the oxyradical scavengers in the lens [44]. Therefore, Trp-deficiency can cause cataract in animal models [8,44,66]. A study in humans also showed an association between the mutation of the LAT2 aromatic amino acid transporter gene used as a Trp transporter and congenital cataract [66]. 

An in vivo study revealed that PRX decreased the incidence of cataract in rats fed on a Trp-free diet [18]. This finding could be due to the beneficial effect of PRX on an increase in GSH level, as well as on the maintenance of S-containing amino acids and water-soluble protein levels [18,47] (Table 3). However, a clinical trial reported that PRX had no effect on the progression of congenital cataract, as indicated by the lack of change of the photographic lens opacity between eyes of an individual to whom PRX was given to one eye and a placebo to another eye [29]. These controversial findings may be due to the different types of cataract. In that study [29], each subject suffered from either complete cataract or nuclear cataract. However, the positive effect of PRX was demonstrated in the cortical type of age-related cataract [12,27,28].

## 5. Safety of Pirenoxine on the Eyes

PRX shows a good safety profile. For example, PRX eyedrops did not delay corneal epithelial wound healing in an in vivo model [67]. The use of PRX was well tolerated during 6 and 24 months of patient treatment, and no adverse events were reported [12,68]. Despite the preservative agents—one of the causes of epitheliopathy—contained in Catalin and Kary Uni, neither corneal epithelial disruption by PRX nor its preservative-adverse effects were observed in a previous study [69]. However, a low prevalence of some adverse events of PRX has been reported, including: (1) conjunctival hyperemia and lacrimation in an animal model [27], and (2) contact dermatitis (proved by patch tests) in humans after a month of PRX instillation [70].

## 6. Conclusions and Future Directions

**Figure 1:**
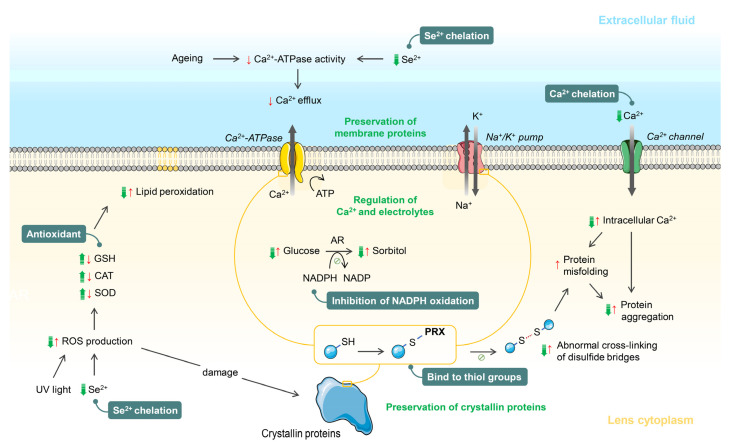
The potential mechanisms utilized by pirenoxine in the prevention of senile cataract and diabetic cataract. Abbreviations: AR: aldose reductase; ATP: adenosine triphosphate; Ca^2+:^ calcium ion; Ca^2+^-ATPase: calcium-adenosine triphosphatase; CAT: catalase; K^+^: potassium ion; Na^+^: sodium ion; NADP/NADPH: nicotinamide adenine dinucleotide phosphate; GSH: glutathione; PRX: pirenoxine; ROS: reactive oxygen species; SOD: superoxide dismutase; UV: ultraviolet (green arrows = effects of pirenoxine, red arrows = changes during cataract formation processes, texts in green and green boxes = mechanisms of pirenoxine).

Most of the current evidence has shown the antioxidant properties of PRX on lens protein protection. It is also an Se^2+^ and Ca^2+^ chelator, an NADPH oxidation inhibitor, and a thiol-group protector, as summarized in Figure 1. Clinically, PRX may decelerate or reverse cortical opacity of the lens; however, it is still inconclusive. The effect of PRX on the changes of physical properties of the lens, including surface shapes, refractive index, and spectral transmission, requires further study. However, it is possible that PRX may alter image forming properties of the lens because PRX can reduce protein aggregation and may reduce cortical opacity of the lens. Both protein aggregation and cortical opacity cause the reduction of light intensity that passes through the lens and an increase in light scattering, leading to poor image quality. Apart from the visual acuity, there are no clinical studies that evaluate the effect of PRX on the improvement of image quality, including glare and contrast sensitivity.

## Figures and Tables

**Table 1:** Effects of pirenoxine on age-related cataract: Evidence from in vitro studies arranged by method of cataract induction.

Induction of Cataract	Source of Lens	Name/Dose/Route/Duration of PRX	Major Findings			Interpretation	Ref
			Lens Opacity	Oxidative Stress	Others		
Ca or selenite (10 mM)	Pig lens homogenate	Pure PRX/0.03, 0.1, and 0.3 μM/ 0–4 d	↓			PRX decelerated Ca- and selenite-induced lens opacification.	[15]
Ca or selenite (10 mM)	Pig lens homogenate	PRX/ 1 μM/5 d	↓			PRX decelerated Ca- and selenite-induced lens opacification.	[16]
Selenite (10 mM)	SD-rat pup lens homogenate	Catalin/0.016, 0.032, 0.080, and 0.1 μM/ 0–4 d Only cataV in Catalin/ 0–4 d	0.016 μM: ⟷ 0.032, 0.080, and 0.1 μM: ↓ (only at d1) ⟷		↓ degradation of water-insoluble lens proteins	High dose PRX decelerated early selenite-induced lens opacification by a deceleration of degradation of water-insoluble lens proteins. CataV in Catalin had no effect on selenite-induced lens opacification.	[15]
Fe^3+^ (10 μM)/ascorbate	Rat lens homogenate	Catalin/ 0.1–1000 μM/2 h		↓ TBA ↓ lipid hydroperoxide		Catalin prevented ROS damage of the lens after induction with Fe^3+^/ascorbate.	[17]
Fe^3+^/ascorbate, Hb (10 μM), fMLP-stimulated macrophages (10 nM)	Rat whole lens	Catalin/ 0.1–1000 μM/2 h		↓ TBA ↓ lipid hydroperoxide		Catalin prevented ROS damage of the lens after an induction with either Fe^3+^/ascorbate, Hb, or stimulated macrophages.	[17]
X (600 μM)/ XO (0.1 U/mL)	Rat whole lens	Catalin/ 0.1–1000 μM/2 h		↓ lipid peroxidation ⟷ Superoxide ⟷ Urate		Catalin prevented ROS damage of the lens with mechanisms other than inhibition of X/XO system.	[17]
UVC (4 h)	Pig lens homogenate	Pure PRX/ 0.1, 1, 10, 100, and 1000 μM/ 0–4 h	PRX (1000 μM): ↓ PRX (<1000 μM): ⟷			Pure PRX and cataV provided comparable benefits in decelerating lens protein opacity via the deceleration of lens degradation. The combination therapy provided greater efficacy than the monotherapy.	[15]
		Catalin/ 16, 32, 80, and 100 μM PRX + cataV/ 0–4 h Only cataV in Catalin/ 0–4 h	↓ ↓		↓ degradation of γ-crystallins ↓ degradation of γ-crystallins		
m-calpain activated by Ca	Pig lens homogenate	Catalin/ 0, 32, 80, and 100 μM Pure PRX/100 μM			⟷ degradation of β- and α-crystallins	Catalin failed to decelerated proteolysis of lens induced by m-calpain.	[15]
UVB (6 h)	Pig lens homogenate	Catalin/ 0.1, 1, 10, and 100 μM/2 h	⟷			Catalin had no protective effect against UVB-induced cataract.	[15]

Abbreviations: <: less than, ⟷: no change/no effect on, ↓: decrease, Ca: calcium, cataV: Catalin-formulated vehicle only, d: day, ELISA: enzyme-linked immunosorbent assay, fMLP: N-formyl methionyl-leucylphenylalanine, GSH: reduced glutathione, Hb: hemoglobin, h: hour, K: potassium, Na: sodium, PRX: pirenoxine, Ref: references, qid: 4 times a day, ROS: reactive oxygen species, Rx: treatment, SD: Sprague–Dawley, SOD: superoxide dismutase, SC: subcutaneous, SPE: single-point energy, TBA: thiobarbituric acid, UVB: ultraviolet-B, UVC: ultraviolet-C, X/XO: xanthine/xanthine oxidase.

**Table 2:** Effects of pirenoxine on age-related cataract: Evidence from ex vivo, in vivo, and clinical studies arranged by type of studies and method of cataract induction.

Study Types	Models	Induction of Cataract	Name/Dose/Route/Duration of PRX	Major Findings			Interpretation	Ref
				Lens Opacity/ Evaluation Time	Oxidative Stress	Others		
Ex vivo	Rabbit lens	Fe^3+^/ ascorbate	Catalin/0.005%, 2 drops q 1 h/topical/8 h daily (total 2 d) before incubation with FeCl_3_		↓ conjugated- dienes ↓ lipid soluble fluorescent compound		Catalin decreased oxidative degradation of lipids in the lens after induction with Fe^3+^.	[17]
In vivo	Rabbit	IVT 50 µM, 100 µM Hb at 2, 4, 6, and 8 d	Catalin/0.005%, 2 drops q 1 h/topical/8 h daily (total 4 d) before IVT Hb		↓ conjugated- dienes ↓ lipid soluble fluorescent compound		Catalin decreased oxidative degradation of lipids in the lens after induction with IVT Hb.	[17]
In vivo	Rabbit	IVT diquat (300 μM)	Catalin/0.005%, 2 drops q 1 h/topical/8 h daily (total 4 d) before IVT diquat		↓ conjugated- dienes ↓ lipid soluble fluorescent compound		Catalin decreased oxidative degradation of lipids in the lens after induction with IVT diquat.	[17]
In vivo	Wistar rat	A single dose of 19 µmol/kg of selenite via SC route at d3	PRX/0.8 mg/15 mL, tid/topical/7 d		Serum: ↑ SOD ↑ CAT ↓ MDA Lens: ↑ SOD ↑ CAT ↑ GSH		PRX increased antioxidative enzymes in both lens and serum after induction with selenite. PRX decreased oxidative degradation of lipids in serum.	[19]
In vivo	Mouse	Senescence-accelerated inbred	Catalin/0.005%, qid/120 d	↓ progression ↓ wedge opacity formation			PRX decelerated progression of age-related cataract.	[28]
In vivo	Dog with age-related incipient cataract	None	PRX/0.05%, 1–2 drops, 3–5 times/d/average 8 mo	↓ opacity or ↓ progression: 72.2% % improvement: Cortical type: 62% Cortical and nuclear type: 30%			PRX reversed opacity and retarded progression of age-related cataract particularly at the cortical region of the lens.	[27]
In vivo	SD-rat pup	A single dose of 19 µmoL/kg of selenite via SC route	Catalin/2.5 and 5 mg/kg single dose/SC/3 d before selenite injection	2.5 mg/kg: ⟷/d 3 ⟷/d 4 5 mg/kg ↓/d 3 ⟷/d 4			Pretreatment with high-dose subcutaneous Catalin only prevented early gross lens opacity in selenite-induced cataract. IVT Catalin also failed to decelerate gross lens opacity.	[15]
			Catalin/ 2 mg/mL single dose/IVT/after selenite injection	⟷/d 5				
In vivo	Wistar rat	A single dose of 19 µmol/kg of selenite via SC route	Catalin solution/0.03%/topical/1 time 1.5 h before selenite injection and qid for 1 wk after selenite injection Catalin liposome/ 0.24 mg/mL (particle size 100 nm)/topical/1 time 1.5 h before selenite injection and qid for 1 wk after selenite injection	By Scheimpflug camera/d 0–7: ⟷ By slit-lamp microscope/ d 1–4: ⟷			Neither solution or liposomal forms of Catalin could prevent or decelerated selenite-induced cataract.	[25]
				By Scheimpflug camera/d 0–7: ⟷ By slit-lamp microscope/ d 1–4: ⟷				
In vivo	Wistar rat lens homogenate	A single dose of 19 µmol/kg of selenite via SC route	Catalin solution/0.03%/topical/1 time 1.5 h before selenite injection and qid for 1 wk after selenite injection		⟷ GSH	⟷ Na/K ratio ⟷ Ca	Neither soluble or liposomal forms of Catalin changed GSH, Na, K, or Ca level of the lens exposed to selenite.	[25]
			Catalin liposome/ 0.24 mg/mL (particle size 100 nm)/topical/1 time 1.5 h before selenite injection and qid for 1 wk after selenite injection		⟷ GSH	⟷ Na/K ratio ⟷ Ca		
Clinical	Patients aged > 40 yr with initial cortical cataract	None	Catalin/24 mo	By slit-lamp microscope: ↓ opacity and ↓ progression/mo3, 6, 12, 18, and 24 (especially in age <59 years and after 18 mo use) % increased opacification • Catalin: 1.425 • Control: 9.228			Catalin decelerated lens opacity and slowed progression of cortical type of presenile and aged-related cataract. The change was more obvious in those younger than 59 years. Peak effect was observed after 18 months of treatment.	[12]
Clinical (double blinded RCT)	Patients with early idiopathic cataract, mean age 60.3 yr (PRX vs. BA, BA vs. control)	None	Catalin/ q 8 h/topical/ 22 mo	% decrease/ q 1 mo *(mo 1–14), mo 18, mo 22:* • Catalin: none • BA: high • Control: none		VA/*1, 2 mo*: • Catalin ↓ • BA ↑ • Control ↓ % operated-eyes/22 mo • Catalin: high • BA: low • Control: high	In age-related cataract BA decelerated or reversed lens opacity, and VA more extensively than PRX. BA also had greater impact on the reduction of the number of cataract operations. From the raw data, PRX seemed not to have effects on lens opacity, VA, and number of cataract operation. (No direct comparison between PRX and control.)	[26]
Clinical (double blinded RCT)	Patients with age-related cataract (<50% extension), age ≥ 40 yr	None	Catalin/6 times/day/topical/24 mo	⟷ progression		⟷ VA	PRX had no effect on early age-related cataract.	[29]

Abbreviations: ⟷: no change/no effect on, ↑: increase, ↓: decrease, BA: benzyl alcohol, CAT: catalase, d: day, F: female, Fe: iron, FeCl_3_: ferric chloride, GSH: reduced glutathione, h: hour, Hb: hemoglobin, IVT: intravitreal, M: male, MDA: malondialdehyde, mo: month, PRX: pirenoxine, q: every, qid: 4 times a day, RCT: randomized controlled trial, Ref: references, Rx: treatment, S: sulfur, SC: subcutaneous, SD: Sprague–Dawley, SOD: superoxide dismutase, tid: 3 times a day, VA: visual acuity, wk: week, yr: year.

**Table 3:** Effects of pirenoxine on diabetic cataract and tryptophan-deficiency models.

Study Types	Source of Lens	Induction of Cataract	Name/Dose/Route/Duration of PRX	Major Findings of the Lens					Interpretation	Ref
				Opacity	GSH	Water-Soluble Protein	S-Containing Amino Acids	Others		
1. Diabetic Cataract										
In vitro	SD rat lens	Hypergalactosemic diet (50% galactose + 50% standard food)	PRX/10^−7^ M, 10^−6^ M, 10^−5^ M, or 10^−4^ M/11–96 h		10^−7^ M: ⟷ 10^−6^ to 10^−4^ M: ↑	10^−7^ M and 10^−6^ M: ⟷ 10^−5^ M and 10^−4^ M: ↑	10^−6^ to 10^−5^ M: ⟷ 10^−4^ M: ↑		Only a high concentration of PRX increased GSH and preserved lens protein by binding to sulfhydryl group.	[16]
In vitro	Wistar rat whole lens	D-galactose (250 mmol/L)	Pure PRX/0.0053%/ 6–24 h	↓ opacity ↓ progression of lens opacity					PRX delayed progression and improved lens transparency.	[29]
In vitro	Rat lens	D-galactose (250 mM)	Catalin/100 μL/24 h	↓ opacity (h 12, h 18, and h 24)					PRX improved lens transparency.	[30]
In vitro	Goat whole lens	Glucose or galactose: 50,100, and 200% over the normal glucose concentration at 0.99 g/L	Catalin/0.001% and 0.01%/7 d	↓ onset of opacity by 12–24 h (effect of 0.001% PRX = 0.01% PRX) ↓ opacity at 12–18 h (effect of 0.001% PRX = 0.01% PRX)					0.001% and 0.01% Catalin delayed the onset of opacity and improved lens transparency.	[31]
In vitro	Cow lens	Sorbitol	Catalin/ 60 µM/48 h					↓ sorbitol	Catalin decreased sorbitol content in lens	[32]
In vivo	SD rat	Hypergalactosemic diet (50% galactose + 50% standard food)	PRX/0.005%, 0.01, or 2%, 2 drops tid/topical/30 d simultaneously with galactose administration	↓ incidence of cataract by 40%	↑	↑	↑		PRX increased GSH and preserved lens protein by binding to the sulfhydryl group. PRX prevented diabetic cataract.	[16]
In vivo	Rat	10 mL/kg of 50% D-galactose bid/IP/90 d + 10% D-galactose water and food/oral/90 d	Catalin/ 0.8 mg/15 mL/topical/3 drops tid/90 d simultaneously with galactose administration	↓ opacity (d 20, d 30, d40, d60 and d90)					PRX improved lens transparency of diabetic cataract.	[30]
In vivo	Wistar rat	10 mL/kg of 50% D-galactose bid/IP/30 d + 10% D-galactose water/oral/ 30 d	Pure PRX/0.0053% tid/topical/60 d after d30 of galactose administration	↓ opacity (10 d–90 d) ↓ progression of lens opacity (10 d–90 d)					PRX delayed progression and reversed lens opacification of diabetic cataract.	[29]
In vivo	Rabbit	Alloxan	Catalin	↓ opacity				↓ Na ↑ K	PRX prevented and delayed lens transparency of diabetic cataract by controlling electrolytes.	[33]
In vivo	Rat	Alloxan 50 mg/kg IV	Catalin/20 mg/kg/IP/daily for 6 wk	100% delayed onset of opacity 81.6% had no lens opacity (wk 5 and wk 6					PRX delayed onset and progression of diabetic cataract.	[65]
In vitro	Rat lens	Glucose 55.5 mM/5 d	PRX/5 d			↑	↑		PRX preserved lens protein by binding to the sulfhydryl group.	[52]
In vivo	Rat	Hypergalactosemic diet	PRX/20 d		↑			↓ aldose reductase activity	PRX increased GSH and decreased aldose reductase activity.	[52]
2. Congenital Cataract										
In vivo	Pigmented rabbit	Tryptophan-free diet (30 d)	PRX/0.005%, 0.01 or 2%, 2 drops tid/topical/30 d	↓ incidence of cataract by 40%/d 30	↑	↑	↑		PRX prevented cataract. PRX increased GSH and preserved lens protein by binding to the sulfhydryl group.	[16]
In vivo	Rabbit	Tryptophan-free diet	PRX/20 d		↑	↑	↑	↓	PRX increased GSH and preserved lens protein by binding to the sulfhydryl group. PRX decreased aldose reductase activity.	[52]
Clinical (double blinded RCT)	Patients with congenital cataract (age 6–8 wk)	None	Catalin/ 6 times/day/topical/16 wk	⟷ progression					PRX had no effect on congenital cataract.	[27]

Abbreviations: =: equal, ⟷: no change/no effect on, ↑: increase, ↓: decrease, AA: amino acids, bid: 2 times a day, d: day, GSH: reduced glutathione, h: hour, IP: intraperitoneal, IV: intravenous, PRX: pirenoxine, Ref: references, S: sulfur, SC: subcutaneous, SD: Sprague–Dawley, tid: 3 times a day, wk: week.
